# Quantum Dot-Based Nanosensors for In Vitro Detection of *Mycobacterium tuberculosis*

**DOI:** 10.3390/nano14191553

**Published:** 2024-09-26

**Authors:** Viktor V. Nikolaev, Tatiana B. Lepekhina, Alexander S. Alliluev, Elham Bidram, Pavel M. Sokolov, Igor R. Nabiev, Yury V. Kistenev

**Affiliations:** 1 Laboratory of Laser Molecular Imaging and Machine Learning, National Research Tomsk State University, 634050 Tomsk, Russia; nikolaev.v@mail.tsu.ru (V.V.N.); tatiana.lepekhina@mail.tsu.ru (T.B.L.); alliluevas@stoptb.tomsk.ru (A.S.A.); 2 Tomsk Phthisiopulmonology Medical Center, Rosa Luxemburg St., 634009 Tomsk, Russia; 3 Department of Biomaterials, Nanotechnology and Tissue Engineering, School of Advanced Technologies in Medicine, Isfahan University of Medical Sciences, Isfahan 81746-73461, Iran; elhambidram@gmail.com; 4 Life Improvement by Future Technologies (LIFT) Center, Skolkovo, 143025 Moscow, Russia; p.sokolov@lift.center; 5 Laboratory of Nano-Bioengineering, Moscow Engineering Physics Institute (MEPhI), National Research Nuclear University, 115409 Moscow, Russia; 6 Department of Clinical Immunology and Allergology, Institute of Molecular Medicine, Sechenov First Moscow State Medical University (Sechenov University), 119146 Moscow, Russia; 7 Laboratoire BioSpecT (BioSpectroscopie Translationnelle), Université de Reims Champagne-Ardenne, 51100 Reims, France

**Keywords:** quantum dot, nanosensor, tuberculosis, diagnostics

## Abstract

Despite the existing effective treatment methods, tuberculosis (TB) is the second most deadly infectious disease, its carriers in the latent and active phases accounting for more than 20% of the world population. An effective method for controlling TB and reducing TB mortality is regular population screening aimed at diagnosing the latent form of TB and taking preventive and curative measures. Numerous methods allow diagnosing TB by directly detecting *Mycobacterium tuberculosis* (*M.tb*) biomarkers, including *M.tb* DNA, proteins, and specific metabolites or antibodies produced by the host immune system in response to *M.tb.* PCR, ELISA, immunofluorescence and immunochemical analyses, flow cytometry, and other methods allow the detection of *M.tb* biomarkers or the host immune response to *M.tb* by recording the optical signal from fluorescent or colorimetric dyes that are components of the diagnostic systems. Current research in biosensors is aimed at increasing the sensitivity of detection, a promising approach being the use of fluorescent quantum dots as brighter and more photostable optical tags. Here, we review current methods for the detection of *M.tb* biomarkers using quantum dot-based nanosensors and summarize data on the *M.tb* biomarkers whose detection can be made considerably more sensitive by using these sensors.

## 1. Introduction

Tuberculosis (TB) is an infectious disease caused by *Mycobacterium tuberculosis* (*M.tb*) and most often affecting the lungs. In 2022, there were 10.6 million TB cases worldwide, including 5.8 million men, 3.5 million women, and 1.3 million children; a total of 1.3 million people (including 167,000 patients with concomitant HIV infection) died from TB. Globally, TB is the second leading cause of death among infectious diseases after COVID-19, with death rate higher than that among AIDS patients [1]. TB usually has particularly severe consequences for women of reproductive age, especially during pregnancy, being among the top five killers of women aged 20–59 years [2]. Recent estimates show that around 1.7 billion people are latently infected with *M.tb* [3]. At the same time, traditional diagnostic methods, such as chest X-ray and TB skin tests, are not sufficiently sensitive and specific to reliably diagnose latent forms of TB [4], especially against the background of other diseases or pathological conditions [5]. The risk of progression of latent TB infection to the active form is estimated at 10% [6]. One of the health-related targets of the United Nations Sustainable Development Goals (SDGs) is to end the TB epidemic by 2030 [1]. To attain this goal, it is necessary not only to combat active forms of TB, but also to detect and treat latent TB, which requires methods for diagnosing *M.tb* infection at the earliest stages.

## 2. Current Tuberculosis Diagnostic Methods

TB can be diagnosed by either directly detecting *M.tb* in a clinical specimen or detecting the biomarkers associated with *M.tb* infection. The numerous methods routinely used for TB diagnosis [7,8,9] can be divided into four major groups: rapid molecular diagnostic tests, cultural methods, provocation tests, and optical diagnostic methods (Table 1). All the procedures except for skin tests are performed in vitro.

### 2.1. Molecular Diagnostic Tests

Polymerase chain reaction (PCR) is a molecular biology technique for amplifying specific DNA fragments to detectable amounts. The amplification involves multiple cycles of heating and cooling causing multiple replications of the DNA fragments. A PCR-based method has been used for detecting specific *M.tb* DNA fragments [10]. This method is effective for TB diagnosis at early stages, when the amount of *M.tb* is insufficient for detection by classical methods. PCR tests also allow analyzing the drug resistance of specific *M.tb* strains. These tests have strict requirements for laboratory room purity and personnel skills because their high sensitivity has a downside: contaminants are likely to be involved in the reaction. PCR tests are suitable for the detection of TB sepsis and disseminated TB, but not for population screening, where they may yield false-negative results.

LF-LAM is a lateral flow urine test for diagnosis of TB through detecting lipoarabinomannan, a mycobacterial cell wall lipoglycan. Its drawback is a low sensitivity [13]. Because lateral flow tests are inexpensive and easy to perform, they are often used for diagnosing TB by detecting IgG antibodies against TB-specific proteins in blood and serum samples [14]. A test for the simultaneous detection of IgG and IgM antibodies has also been developed [25]. In this case, the test band contained a mixture of recombinant TB antigens that ensured a diagnostic sensitivity of 94.4% and a diagnostic specificity of 98.3%.

Loop-mediated isothermal amplification (LAMP) uses special primers to amplify DNA fragments that form loop-shaped intermediates of different sizes. These fragments can be subsequently detected using fluorescence measurements or agarose gel electrophoresis [26]. The WHO recommends LAMP as a replacement for microscopy in the diagnosis of pulmonary TB [27].

Xpert MTB/RIF Ultra is an improved version of the Xpert MTB/RIF test [28,29]. Xpert MTB/RIF Ultra (in vitro) and Truenat can identify mutations of the *rpoB* gene associated with rifampicin resistance [12,28,29,30]. Xpert MTB/RIF Ultra and Truenat have a higher sensitivity and a shorter time of analysis than conventional PCR tests.

Serological tests detecting antibodies against specific antigens of the recombinant *M.tb* complex have a high specificity but variable sensitivity [14]. However, the sensitivity can be increased by the combined use of tests based on different *M.tb*-complex antigens [14]. It should be noted that these methods may yield false-positive results because specific antibodies may occur in blood long after the recovery from TB.

### 2.2. Tuberculosis Tests Based on T-Cell Analysis

An interferon-gamma release assay (IGRA) is a group of in vitro tests that estimate the release of interferon-gamma (INF-γ) by human immune blood cells (T-cells) [15]. Two blood tests based on this principle have been approved by the FDA: QuantiFERON-TB Gold Plus (QFT) and T-SPOT.TB (T-SPOT). The QFT test is a whole-blood-based enzyme-linked immunosorbent assay (ELISA) measuring the amount of IFN-γ produced in response to two *M.tb* antigens (ESAT-6 and CFP-10). The T-SPOT test measures the number of T-cells that produce INF-γ after stimulation with ESAT-6 and CFP-10. These methods may also yield false-positive results, because T-cells with a “memory” of *M.tb* infection may be retained in the body for long periods of time.

### 2.3. Culture Methods

Culture methods remain the gold standard of TB diagnosis confirmation. In this case, biological material is placed onto a solid or liquid differential diagnostic nutrient medium stimulating the growth of mycobacterial colonies. Several culture methods are currently used: acid-fast mycobacteria (AFB) [16], BАСТЕС (usually with MGIT 460 or MGIT 960) [31], and BacT/ALERT 3D [18].

The AFB method is unpopular because of the extremely long time of analysis, the latest experimental study that used it dating back to 1997 [31,32]. In this method, human sputum or other biological material is stained for acid-fast bacteria. The sample to be tested is inoculated into a specific growth medium and inserted into the instrument for incubation and periodic fluorescent reading. Each vial contains a chemical sensor detecting an increase in the amount of carbon dioxide produced by the growing microorganisms. The instrument monitors the sensor every 10 min for an increase in its fluorescence, which is proportional to the amount of CO_2_, a positive reading indicating the presence of viable microorganisms.

BACTEC is a fully automated system not only for *M.tb* detection, but also for the analysis of *M.tb* sensitivity to all first-line drugs, including pyrazinamide. BACTEC is a reference method with high sensitivity and specificity, but it takes around 10 days to obtain the result [33]. BacT/ALERT 3D allows automated monitoring of microorganism growth in the culture medium by estimating the CO_2_ release, which is measured by the increase in reflectance. It has a high sensitivity with a short culturing time. BacT and BACTEC have similar operating principles but differ in the details of technology and design. The key limitation of all these methods is a too long time of analysis.

### 2.4. Skin Tests

In vivo tuberculin skin tests are based on the provocation of the body immune response by TB-associated molecules [19]. For example, the Mantoux test uses a tuberculin solution injected intradermally. All of these tests suffer from frequent false-positive and false-negative results. The point is that the immune system responds to tuberculin if there are mycobacteria in the body, and most people receive the bacteria in the form of the BCG vaccine soon after birth. Recently, the WHO included Diaskintest, which is an advanced and more accurate variant of the Mantoux tuberculin test [34], into the list of recommended skin tests for TB.

### 2.5. Tests Based on Mycobacterium Staining

Staining methods identify specifically stained acid-fast mycobacteria, actinomycetes, and other acid-fast microorganisms by means of optical microscopy. These methods differ in the staining solution used, which determines the sensitivity and specificity of analysis. The weak point of this group of TB tests is a complex procedure of analysis that requires considerable time and highly skilled personnel.

### 2.6. Other Methods

Chest X-rays are commonly used in TB diagnosis. They can help to identify abnormalities in the lungs that suggest TB infection, such as nodules, cavities, or infiltrates. However, it should be noted that chest X-rays alone cannot definitively diagnose TB.

Matrix-assisted laser desorption/ionization–time-of-flight mass spectrometry (MALDI-TOF MS) is based on an ionization technique that allows the ionization of biological macromolecules, such as peptides, proteins, DNA, oligonucleotides, and lipopolysaccharides, in the presence of a special matrix under laser irradiation [35]. Wang et al. [36] evaluated MALDI-TOF MS as a means of *M.tb* nucleic acid detection for the rapid diagnosis of TB and estimation of *M.tb* drug resistance. The effectiveness of MALDI-TOF MS can be improved by using the protocol of *M.tb* cell destruction and protein extraction [37].

Liquid chromatography–tandem mass spectrometry (LC-MS/MS) is based on coupling mass spectrometers in series to analyze complex mixtures [38]. For example, liquid–liquid extraction and LC-MS analysis were used to determine the pretomanid concentrations in human plasma [39]. A clinical trial involving TB patients has demonstrated that this is a reliable and reproducible method of pharmacokinetic analysis. Another study used the LC-MS technique to detect specific *M.tb* peptides in mouse blood serum. Sixty-five peptides from four recombinant *M.tb* proteins were identified in the mouse blood [40]. This method does not directly detect *M.tb*, but it is useful in the monitoring of TB treatment [41].

**Figure 1:**
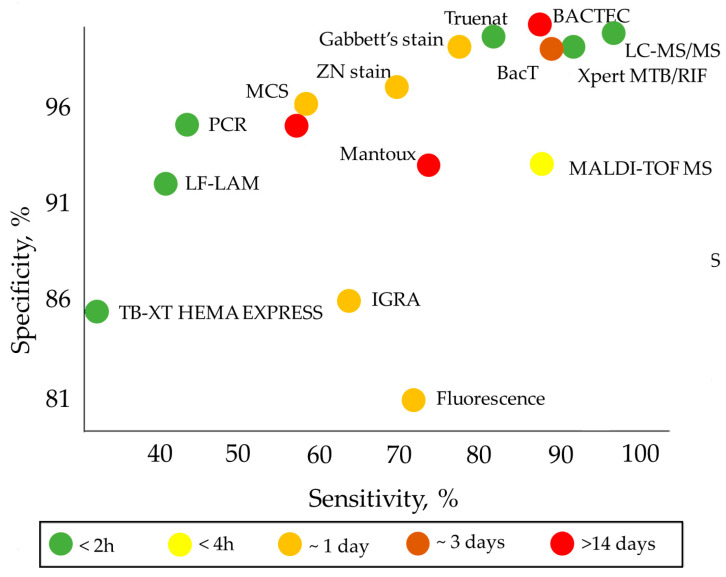
Sensitivity and specificity of tuberculosis diagnostic methods shown in Table 1.

Figure 1 graphically illustrates the key data from Table 1.

Scrutiny of the above TB diagnostic methods shows that none of them are free from drawbacks and limitations. Therefore, development of new simple and effective methods of TB diagnosis is an urgent task.

## 3. Quantum Dot-Based Nanosensors for *M. tuberculosis* Detection and Tuberculosis Diagnosis

Most of the above methods, be it PCR-based molecular detection methods using fluorescent probes, lateral flow tests using colloidal gold nanoparticles or colored latex microparticles, ELISA and ELISPOT tests, tests for CO_2_ accumulation, or specific *M.tb* staining, involve the detection of an optical signal. Traditionally, all commercial products for *M.tb* detection and TB diagnosis use organic fluorescent or colorimetric dyes, which have recently been increasingly replaced with fluorescent quantum dots (QDs) [42].

QDs are inorganic semiconductor nanocrystals 2–10 nm in size with a high fluorescence quantum yield due to a high molar absorption coefficient and a high efficiency of internal conversion of the absorbed photon energy into fluorescence [43]. Another benefit of QDs is their extremely long luminescence lifetime compared to fluorescent biomolecules. This allows time-resolved detection with an increased signal-to-noise ratio, which enhances the detection sensitivity [44,45]. The narrow emission peak and wide absorption spectrum make it possible to excite QDs of different colors with a single broad-spectrum source and perform multiplexed detection. QDs have a semiconductor core (CdSe, CdS, CdTe, InP, InAs, AgInS_2_, CuInS_2_, PbSe, etc.), often coated with a shell to passivate the surface trap states and protect the core from aggressive environments and photo-oxidative degradation, as well as to meet biosafety requirements [46,47,48]. The use of QDs in biosensors implies their contact with biological fluids, which requires solubilized, biocompatible QDs. Therefore, the development of efficient methods for QD solubilization in water and biological fluids is crucial for their full potential to be used in this field. There are two main approaches to obtaining water-soluble QDs. The first is the synthesis of QDs coated with hydrophobic ligands in organic solvents followed by replacement of the hydrophobic ligands with hydrophilic ones [49]. The second approach is obtaining originally hydrophilic QDs, e.g., their synthesis in a reverse microemulsion system, where an aqueous solution is dispersed in an organic phase [50]. However, the latter methods are usually more complicated. In addition, published methods of direct QD synthesis in the aqueous phase [51] do not ensure sufficient control of the QD size, shape, and, hence, optical characteristics, and most of them require additional procedures for modifying the QD surface to make them more stable in aqueous solutions. The hydrophilic ligands used for the direct synthesis of water-soluble QDs or for the replacement of original hydrophobic ligands include mercaptopropionic acid [52], thioglycolic acid [53], glutathione [54], cysteamine hydrochloride [55], D,L-cysteine [56], and some others. The ligands vary in size, charge, and isoelectric point, but most of them contain a thiol group, which serves to bind with the inorganic epitaxial shell of QDs. The ligands not only make QDs water-soluble and protect them from potentially aggressive environments, but also ensure the preservation of the QD fluorescence properties in aqueous solutions and biological fluids [48,57,58]. In addition, the surface ligands are used for conjugating the QDs with biological recognition (or capture) molecules, such as antibodies or their fragments [59], single-domain antibodies [60], aptamers [61], and proteins [62]. Usually, they are conjugated via the formation of permanent covalent bonds, e.g., by carbodiimide chemistry methods [62] or by using glutaraldehyde [63], but noncovalent binding is also possible, e.g., by using pairs of affinity molecules, such as streptavidin and biotin [64].

**Figure 2:**
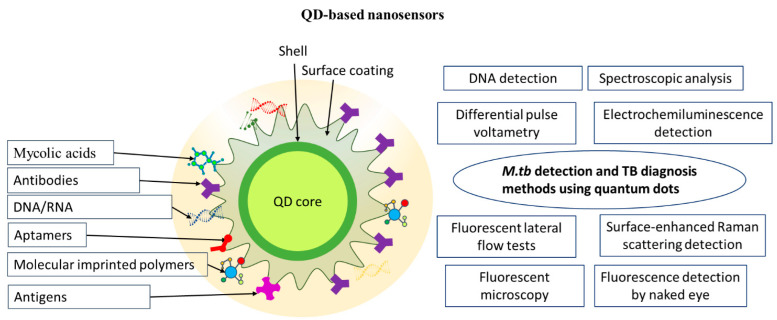
Schematics of a quantum dot-based nanosensor. Abbreviations: *M.tb*, *M. tuberculosis*; TB, tuberculosis.

A rise in temperature can also lead to deterioration of the QD optical properties, which occurs, e.g., when they are used in solar batteries or other electronic devices [65,66]. QD thermostability can be increased by incorporating QDs into hydrophilic glass shells or polymer shells [67]. However, biosensors usually operate at room temperature, so that thermal degradation is not an issue. Functionalization of the QD surface, e.g., with cysteamine, makes it possible to use QDs as reporter fluorescent tags in loop-mediated isothermal amplification, i.e., the optical and colloidal characteristics of QDs can remain stable at temperatures of around 60 °C [68]. Figure 2 shows a typical structure of QD-based nanosensors.

An ideal QD-based fluorescence nanosensor should combine a bright fluorescent label and a highly specific capture molecule [69]. This capture molecule can be a protein (e.g., an antibody or recombinant antigen), peptide, oligonucleotide, etc. [60,70]. After the QD-based nanosensor has bound the target biomolecule, the QD fluorescence signal can be detected and quantified [71,72,73]. Numerous methods for covalent and noncovalent conjugation of ligands to the QD surface (e.g., electrostatic interaction and metal ion chelation) have been developed [42,59,74]. The possibility of using multiple QDs with different emission spectra enables the simultaneous detection of several biomarkers, which increases the diagnostic accuracy [75,76,77]. An additional advantage of this technology is the stability of fluorescence properties during storage [78].

**Figure 3:**
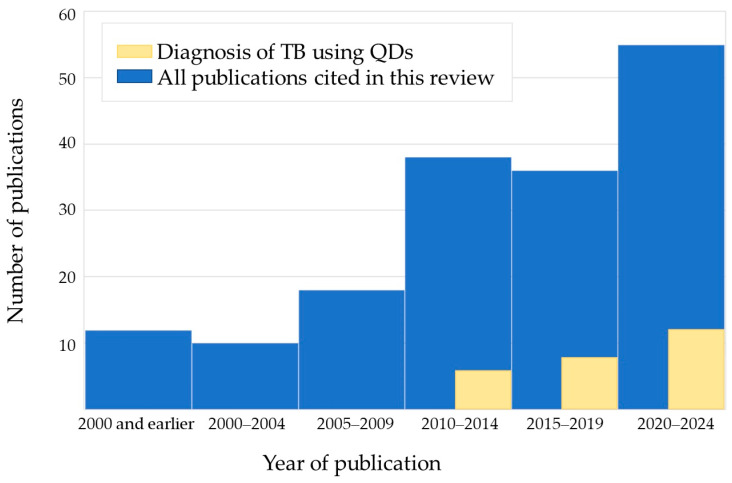
Numbers of analyzed publications by year. Abbreviations: TB, tuberculosis; QDs, quantum dots.

A total of 28 articles retrieved by the keywords *quantum dot*, *tuberculosis*, and *Mycobacterium tuberculosis* and 43 articles retrieved by the keywords *quantum dot* and *tuberculosis* have been found in the PubMed database. Of these publications, 37 deal with TB diagnosis using QD-based nanosensors, 18 of them published in the past five years (including six reviews published in the past four years [7,8,9,79,80,81,82]). The number of these publications by year is shown in Figure 3. In total, 170 articles are cited in this review, 124 of them published in the past 10 years.

The methods of *M.tb* detection and TB diagnosis using QD-based nanosensors are shown in Table 2.

Not all of the biomarkers described above are completely specific, because their occurrence may be related to concomitant diseases, body conditions, etc. Currently, there is no biomarker or combination of biomarkers that allows diagnosing active forms of TB with an accuracy close to 100%. Thus, the search for a combination of biomarkers with a high specificity is an urgent task. New potential *M.tb* biomarkers that can be detected by new QD-based fluorescent nanosensors are listed in Table 3.

Host transcript RNA/DNA signatures are a group of biomarkers associated with the host gene expression in response to *M.tb* infection. For some markers listed in Table 3, there are suitable QD-based nanosensors shown in Table 2: *GBP2* [83,106], *GBP5* [83,106], *GBP6* [83,106], *IS6110* gene [96], *rpoB531* gene [91], and *katG315* gene [91]. Regarding *PRDM1*, it is also associated with lymphoma [135]. To date, there is no QD-based nanosensor for arginase 1 detection. The group of acids and their derivatives consists of two important TB biomarkers: MN [100] and MAs [88,89]. For both markers, sets of QDs and conjugates that can be used for TB diagnosis are shown in Table 2. The group of enzyme biomarkers includes enzymes of three types that could be used for TB diagnosis: MNAzymes, ADA, and KatGs. To date, QDs functionalized with MNAzymes [87] have been proposed as TB diagnostic agents. Regarding KatGs, there are methods for detecting the encoding genes, but there are no biosensors for detecting the enzymes themselves. No nanosensors for ADA detection have been reported to date.

The groups of specific surface protein and mycobacterial antigen biomarkers can be pooled because both include specific proteins and other antigens associated with *M.tb.* To date, three main protein antigens from this group have been studied in terms of TB diagnosis using nanosensors: CFP-10, ESAT-6, and Ag85B [90,97,99,105].

## 4. Multiple Diagnostic Markers for *M. tuberculosis* Detection and Tuberculosis Diagnosis

Accurate diagnosis of TB or detection of *M.tb* often requires the simultaneous detection of several biomarkers, in particular, when it is necessary to discriminate between TB and other infectious diseases, to determine the stage of TB, or detect drug resistance. For example, the detection of cytokine IFN-γ alone usually has a low diagnostic value because its level is affected by numerous factors [136]. Wang et al. [137] developed a multiplexed flow cytometry kit based on fluorescently labeled microbeads and capture molecules for the detection of 16 TB biomarkers. They found that the detection of IFN-γ, IFN-γ inducible protein-10 (IP-10), monokine induced by IFN-γ (MIG), TNF-α, and IL-2 revealed distinct differences between patients with active-phase TB and healthy subjects. On the other hand, the sensitivity and specificity of active-phase TB diagnosis based on the detection of IP-10 or MIG alone were comparable to those in the case of IFN-γ detection. Combined detection of IFN-γ, IP-10, and MIG considerably enhanced the sensitivity and specificity compared to the detection of individual cytokines and chemokines. La Manna et al. [138] used the analyzed 48 cytokines and chemokines by means of the Luminex Bead Array Multiplex Immunoassay for precise discrimination of TB from other pulmonary diseases. They found that the IL-3, IL-12-p40, LIF, IFNα2, IL-2ra, IL-13, b-NGF, SCF, TNF-β, TRAIL, IL-2, IFN-γ, IP-10, and MIG levels were considerably higher in patients with active and latent TB forms compared to non-TB patients, whereas the MIF level was considerably lower in patients with active TB compared to patients without TB and with latent TB. The combination of seven biomarkers made it possible to diagnose active and latent phases of TB with an accuracy of 88.89 and 82.35%, respectively, and identify non-TB patients with an accuracy of 90%. In these studies, multiplexed detection was performed by means of flow cytometry using microbeads fluorescently encoded with organic dyes. Earlier, we demonstrated that microbeads encoded with fluorescent QDs could be used for the same multiplexed analyses that were performed using the commercially available Luminex xMAP^®^ technology [139]. Moreover, the performance of QD-based detection systems was better because QDs have wider absorption spectra, narrower fluorescence spectra [140], and a larger Stokes shift [141] than organic fluorescent dyes.

## 5. Summary and Outlook

Despite substantial advances in the diagnosis and treatment of TB, early diagnosis remains crucial for controlling its spread [142]. This requires the detection of latent infection in *M.tb* carriers who do not manifest active symptoms and those at increased risk of progression from latent to active disease.

Despite logistical problems in low- and middle-income countries, IGRA tests, as well as skin tests, have been approved by the WHO [143]. Their main shortcoming is the low accuracy of the assay.

Most in vitro tests for TB infection assessing the cellular immune response by quantitative or qualitative estimation of IFN-γ release after stimulation with *M.tb*-specific antigens have some limitations [144]. First, these tests require an incubation period of 16–24 h, which precludes same-day diagnosis. Second, in order to keep the cells viable, blood samples should usually be processed within 16 h (at most, 48 h) after collection, and they should be stored in a refrigerator [145,146]. This requires a well-developed laboratory service, with climate and geographical conditions of sample transportation taken into account, although, e.g., T-SPOT.TB allows samples to be stored at room temperature for up to 54 h provided that an additional test kit is used [147,148]. In addition, diagnostic errors caused by uncontrollable factors, inaccurate pipetting, and manipulation errors during centrifugation, decantation, and washing are very likely [149,150]. IGRA requires phlebotomy, which is challenging in pediatric patients [151]. In addition, these tests cannot determine the drug resistance profile of the infectious *M.tb* strains; they are limited to assessing the immune response and distinguishing active TB from latent TB.

Traditional molecular diagnostic methods based on PCR are widely used in the clinic, but only as a supplement to phenotypic detection of *M.tb* and testing of the response to anti-TB drugs [152]. Laboratory sample processing entails the risk of sample contamination with exogenous nucleic acids, which leads to false-positive results. In addition, residual nucleic acids from dead *M.tb* interfere with the correct clinical interpretation of the results of diagnosis and, hence, the choice of treatment [153]. PCR tests are limited to the detection of nucleic acids, the numerous other *M.tb*-specific substances remaining unexplored [154]. The GeneXpert MTB/RIF assay, simultaneously detecting *M.tb* and rifampicin resistance, has been a breakthrough in TB diagnosis, but its use is limited because of its high cost and the need for a constant and stable power source to operate the instrument [155,156]. The assay is also insufficiently sensitive to states with a low *M.tb* content of the sample [157,158]; moreover, the *M.tb* isolates that have silent mutations do not bind to wild-type sequences, which leads to false-positive results when drug resistance is estimated [159]. The lack of information on the full spectrum of mutations associated with drug resistance and the limited reliability in detecting the resistance to TB drugs are the main obstacles to the use of PCR molecular diagnosis [153]. Furthermore, the need for using sputum samples and the long processing time prevent the use of these tests for rapid screening in communities with a high burden of TB [160]. Thus, although PCR-based diagnostic tools are highly sensitive and can detect multiple cases of drug-resistant TB, their practical application is limited.

Although QD-based tests cannot completely replace PCR tests in all aspects (especially where high specificity and sensitivity to specific genetic sequences are required), their use as an alternative or supplement can significantly simplify diagnostic procedures in various fields, including medicine and biotechnology, and reduce their cost [157].

Apart from QDs, other nanomaterials, such as nanoparticles of noble metals, crystals, films, and magnetic nanoparticles, can serve as components of nanosensors solving similar tasks. These are not only nanoparticles of various types [114,115,116], but also, e.g., graphene [112] and graphene-like 2D-materials (trans-graphenes) [132,161,162,163,164,165,166,167]. However, the advantage of QDs is that they act as fluorescent labels, in which capacity they are beyond competition due to their extremely high quantum yield. On the other hand, noble metal nanoparticles, for example, can be used in chemical sensors because excitation induces surface plasmon resonance on them, with the resonance frequency depending on the environment. Thus, these approaches do not compete, but rather complement each other.

QDs have already established themselves as promising constituent elements of biosensors providing a higher sensitivity and specificity of detection than routinely used assays and allowing the development of multiplexed assays for early, more detailed detection of *M.tb* and diagnosis of TB. Despite the undoubtedly high potential, several challenges need to be addressed before the widespread use of QD-based nanosensors for TB diagnosis, such as the search for new suitable conjugates and available highly specific biomarkers, standardization and validation of diagnostic protocols, and advanced cost- and time-reducing solutions. The issue of quantum dot toxicity is still under debate and requires further research [168,169]. However, the data reviewed here show that the unique properties of QDs make QD-based nanosensors promising candidates for biosensing applications, including in vitro TB diagnosis. The use of QDs makes it possible to increase the sensitivity and shorten the time of analysis, which is important for point-of-care diagnosis and wider coverage of diagnostic procedures. The possibility of excitation of QD fluorescence in a wide range of wavelengths and a long fluorescence lifetime allow alleviating the requirements for fluorescence detectors and, hence, the cost of their manufacture, as well as designing more compact devices for reading the fluorescent signal. This would ensure a wider use of these tools in diagnostic practice, thus decreasing the morbidity and mortality from TB. Given the possibility of scaling their production and the low cost of implementation without the requirement for specialized expensive devices, the use of this technology in clinical practice looks highly promising.

The main tasks to be handled by the developers of diagnostic test systems and biosensors for *M.tb* detection and TB diagnosis are an increase in the sensitivity of diagnosis of active and latent forms of TB, their effective discrimination from other pulmonary infections, detection of drug resistance, and reduction in the cost and time of analysis, as well as mass implementation of new solutions into the existing diagnostic practice. First of all, it is necessary to develop new multiplexed panels for the detection of multiple biomarkers, which will increase the specificity of detection and, hence, the accuracy of diagnosis. As shown in our review, panels of five or more biomarkers have already been shown to effectively discriminate various pulmonary infections, mainly in flow cytometry tests using microbeads optically encoded with organic fluorescent dyes. The use of QDs instead of organic fluorescent dyes expands the possibilities of multiplexed testing, because the fluorescence of QDs of different colors can be excited using a single light source and the QD fluorescence peaks are narrower, with a larger Stokes shift and brighter fluorescence. The current limited use of QDs in test systems for in vitro diagnosis is primarily due to the toxicity of heavy metal–based QDs. However, in the past ten years, methods for synthesizing low-toxic QDs have been rapidly developed, and several clinical studies on their use in diagnosis are already underway [170]. Currently, there are many approaches to obtaining solubilized biocompatible QDs that can be conjugated with biological capture molecules in various ways. Numerous papers describe the development of QD-based biosensors and demonstrate their advantages in the sensitivity and specificity of biomarker detection. Some of the sensors are compatible with the available equipment, such as flow cytometers, fluorescence microplate readers, or microarray scanners; others do not require additional expensive equipment, as in the case of immunochromatographic tests (lateral flow tests). The implementation of these developments into diagnostic practice is a matter of the near future. It should also be noted that the possibility of exciting QDs of different colors from a single light source reduces the cost of manufacturing equipment for their detection, because there is no need for different excitation sources, filters, and collimators. The high brightness of the fluorescence signal allows less expensive detectors to be used. Thus, QD-based nanosensors could help to solve not only medical problems, such as the increase in the specificity and sensitivity of detection, but also economic ones, due to the reduced cost of equipment for fluorescence detection.

## Figures and Tables

**Table 1:** The main groups of routine clinical methods for tuberculosis diagnosis.

Assay	Biomaterial Analyzed	Time of Analysis	Advantages	Drawbacks	Sensitivity, Specificity	Ref.	Comment
**Molecular Diagnostic Tests**							
Polymerase chain reaction (PCR)	Serum, urine, blood, sputum, saliva, lung biopsy specimens, BALF, pleural fluid	4–5 h	High specificity; short time of analysis; high informativeness	High cost; limited availability; low sensitivity for non-respiratory specimens	Sensitivity: 47% (42–51%) Specificity: 95% (93–97%) CrI: 95%	[10]	The sensitivity and specificity are averaged results of 9 studies on 709 subjects
Xpert MTB/RIF Ultra	Raw sputum or concentrated sediment	1.5 h	Detection of specific *rpoB* gene mutations associated with rifampicin resistance	High cost	Sensitivity: 89% (85–92%) Specificity: 99% (98–99%) CrI: 95%	[11]	The sensitivity and specificity are averaged results of 22 studies on 8998 subjects, 2953 of them with confirmed TB
Truenat	Raw sputum	1 h	A portable, chip-based, battery-operated device. Suitability for poorly equipped laboratories	Lower accuracy compared to Xpert MTB/RIF Ultra	Sensitivity: 80% (70.2–88.4%) Specificity: 98% (94.5–99.6%)	[12]	The sensitivity and specificity have been estimated in a sample of 250 subjects
LF-LAM	Urine	0.5 h	High efficiency; ease of use; low cost; simple technology; no special equipment required. Detection of TB in subjects for whom other diagnostic methods cannot be used (e.g., HIV patients)	Lower sensitivity compared to Xpert MTB/RIF (though it is higher compared to microscopy methods). Suitability for a limited group of patients. Inability to distinguish *M. tb.* from other mycobacteria, which requires using other diagnostic methods along with the test	Sensitivity: 45% (29–63%) Specificity: 92% (80–97%) CrI: 95%	[13]	The sensitivity and specificity are averaged results of 5 studies on 2313 subjects, 35% of them with confirmed TB
TB-XT HEMA EXPRESS	Blood, serum	0.5 h	Short time of analysis; relatively low cost	Low sensitivity; suboptimal performance in the case of high TB prevalence	Sensitivity: 31% (3.9–78%) Specificity: 85% так (52–93%)	[14]	The sensitivity and specificity have been estimated in a sample of 1386 subjects, 290 of them with confirmed TB
**TB tests based on T-cell analysis**							
IGRA, (T-SPOT.TB, QuantiFERON-TB Gold (QFT))	Blood, serum	Up to 2 days	Insensitivity to previous BCG vaccination or contact with atypical mycobacteria; high efficiency. One-time tests. T-SPOT.TB is less susceptible to immunosuppression than other TB tests and is preferable for patients with HIV or autoimmunity patients under immunosuppression treatment; it can be used before the start of therapy with biological drugs	Low specificity and sensitivity; high cost; inability to distinguish between the active and latent forms of TB; unsuitability as a primary diagnostic test for LTBI or active TB. The bacterium itself is not detected. The result depends on the state of the patient’s immune system	**QFT** Sensitivity: 66% (47–81%) Specificity: 87% (68–94%) **T-SPOT** Sensitivity: 60% (48–72%) Specificity: 86% (65–95%)	[15]	The sensitivity and specificity have been estimated in a sample of 6525 HIV-positive patients, 3467 of them with confirmed TB, including 806 with LTBI and 2661 with active TB
**Culture methods**							
BBL Septi-Chek AFB	Sputum	Up to 23 days	Higher *M.tb* growth rate compared to methods using an isolated dense medium	Low sensitivity; long time of analysis	Sensitivity: 73% Specificity: 93%	[16]	The sensitivity and specificity have been estimated in a sample of 274 specimens
BАСТЕС (MGIT 460 and MGIT 960)	Sputum	Up to 14 days	Rapid identification of *M.tb* and its drug sensitivity. Accelerated testing of all first-line drugs	High cost, justified only for large laboratories. Semi-automatic monitoring of bacterium growth requiring many labor-intensive operations. Use of radioisotopes and the need for disposal of radioactive waste. Long time of analysis	**MGIT 960** Sensitivity: 81.5% Specificity: 99.6% **MGIT 460** Sensitivity: 85.8% Specificity: 99.9%	[17]	The sensitivity and specificity have been estimated in samples of ~8000 clinical specimens per year. The number after MGIT is the number of wells in the plate.
BacT/ALERT 3D	Sputum	24–72 h	Detection of *M.tb* growth; detection of *M.tb* and fungi in blood cultures. Full automation; no radioactive waste	Long time; high cost	Sensitivity: 87.80% Specificity: 99.21%	[18]	The sensitivity and specificity have been estimated in a sample of 2659 clinical specimens
**Skin tests**							
Tuberculin skin tests, Mantoux tests, and Diaskintest (in vivo)	Skin tests	72 h	Availability; low cost; ease of use	Low specificity and sensitivity; unsuitability for diagnosing active TB forms. False-positive results in subjects previously infected with *M.tb*, because their memory T-cells still secrete interferon. Inability to distinguish between the active and latent forms of TB	Sensitivity: 59% Specificity: 95%	[19]	The sensitivity and specificity have been estimated in a sample of 643,694 US Navy recruits
**Tests based on mycobacterium staining**							
Gabbett’s stain, Ziehl–Neelsen stain, modified cold stain (MCS)	Sputum	~24 h	Simplicity; short time of analysis; ease of use; low cost	Low sensitivity and specificity; suitability for pulmonary tuberculosis only; inaccuracy in children and subjects with HIV; multistage and complex procedure. Inability to distinguish between different mycobacteria	**Gabbett’s stain** Sensitivity: 77% Specificity: 98% **Ziehl–Neelsen stain** Sensitivity: 70% Specificity: 97% **MCS** Sensitivity: 60% Specificity: 96%	[20]	The sensitivity and specificity have been estimated in a sample of 100 patients
Fluorescence microscopy	Sputum	~24 h	Short time of analysis; ease of use; specificity	High cost; frequent burn-out of expensive mercury vapor lamps; need for continuous power supply; need for a dark room	Sensitivity: 72% Specificity: 81%	[21]	The sensitivity and specificity have been estimated in a sample of 426 patients
**Other methods**							
X-ray	Radiographic test	1 h	Short time of analysis	High cost; low specificity	Sensitivity: 96% Specificity: 46%	[22]	The sensitivity and specificity are averaged results of 13 studies
MALDI-TOF MS	BALF, sputum	2.5 h	Short time of analysis; reliability; high cost efficiency	Sample preprocessing is required to generate high-quality proteomic profiles, especially for proteins/peptides or other low-abundance analytes in which MS spectra are obscured by more abundant or higher-molecular-weight species. Low specificity because of the noise caused by matrix proteins	Sensitivity: 83% Specificity: 93% CrI: 95%	[23]	The sensitivity and specificity have been estimated in a sample of 214 patients
LC-MS/MS	Urine, blood	1 h	Proteomic analysis of urine; identification of proteins characteristic of TB with high molecular specificity and sensitivity; simultaneous diagnosis of HIV-1 and TB using a blood sample. Structural identity of individual components	Changes in ionization efficiency in the presence of not only proteins, phospholipids, and salts, but also reagents and contaminants	Sensitivity: 94% Specificity: 100%	[24]	The sensitivity and specificity have been estimated in a sample of 57 patients

Abbreviations: LF-LAM, lipoarabinomannan lateral shift test; LTBI, latent tuberculosis infection; MGIT, mycobacteria growth indicator tube; IGRA, interferon-gamma release assay; CrI, credible interval; BALF, bronchoalveolar lavage fluid; MALDI-TOF MS, matrix-assisted laser desorption ionization—time-of-flight mass spectrometry; LC-MS, liquid chromatography—tandem mass spectrometry; MS, mass spectrometry.

**Table 2:** Quantum dot-based nanosensors for *M. tuberculosis* detection and tuberculosis diagnosis.

No.	Biomaterial Analyzed	Biomarker	Capture Molecule	Nanosensor	Method of Detection	Wavelength, nm (Where Relevant)	LOD	Ref.
1	Blood	*TMCC1*, *GBP6*	Oligonucleotides specific for *M.tb* mRNA biomarkers	QD655 and QD525 conjugated with the capture molecules	Toehold-mediated strand displacement with fluorescence quenching by FRET	Emission: 525 Emission: 655 Excitation: 480	GBP6: 1.6 nM TMCC1: 6.4 nM	[83]
2	Blood	IFN-γ	Anti-human IFN-γ antibodies	CdS QDs coupled to magnetic beads conjugated with the capture molecules. Sandwich-type sensor is fabricated on a glassy carbon electrode coated with a well-ordered gold nanoparticle monolayer, which offers a solid support to immobilize the capture molecules	Square-wave anodic stripping voltammetry for quantifying the metal cadmium, which indirectly reflects the amount of the analyte	N/A	0.34 pg/mL	[84]
3	Serum	IFN-γ	IFN-γ aptamer	Gold electrode coated with L-cysteine-SnTeSe QDs functionalized with the capture molecules	Electrochemical impedance spectroscopy detection of the change in the electron transfer resistance upon IFN-γ binding	N/A	0.151 pg/mL	[85]
4	Serum	IFN-γ, TNF-α, IL-2	Antibody pairs for IFN-γ-, TNF-α and IL-2	Sandwich immunoassay sensor consisting of luminol and carbon and CdS QDs integrated with gold nanoparticles and magnetic beads functionalized with the capture molecules, as well as the same capture molecules separately immobilized in three spatially resolved areas of a patterned indium tin oxide electrode to capture the corresponding triple latent TB biomarkers	Electrochemiluminescence detection	N/A	1.6 pg/mL	[86]
5	Sputum	DNA *IS1081*	Specific DNA nanobeacon	QD-based nanobeacon fluorescence probes containing QDs and black hole quenchers. After the target DNA hybridizes with the nanobeacon, the nanobeacon is cleaved into two DNA fragments, and the QDs fluoresce upon moving away from the black hole quenchers	Fluorescence detection by naked eye	Excitation: 280 Emission: 330	3.3 amol/L (2 copies/μL)	[87]
6	N/A	Anti-MA antibodies	MAs	Graphene QDs covalently functionalized with MAs as detection tags for anti-MA antibodies	Fluorescence detection (fluorescent lateral flow assay)	Excitation: 360 Emission: 470	N/A	[88]
7	N/A	Anti-MA antibodies	MAs	CdSe/ZnS QDs covalently functionalized with MAs as detection tags for anti-MA antibodies	Fluorescence detection (fluorescent lateral flow assay)	Excitation: 390 Emission: 474	N/A	[89]
8	Pure CFP-10 solution	CFP-10	Pair of anti-CFP-10 antibodies (G2 and G3)	Glass slide coated with magnetoplasmonic core/shell nanoparticles (Fe_3_O_4_/Au) functionalized with G2. Graphene QDs functionalized with conjugate of gold-binding protein with G3. Upon binding of CFP-10 by a G2–G3 sandwich, immunoassay is formed	Dual metal-enhanced fluorescence and surface-enhanced Raman scattering detection	Excitation: 320 Emission: 436, 516	0.0511 pg/mL	[90]
9	Pure DNA	*rpoB531*, *katG315*	ssDNA specific for target DNA	QD_535_ and QD_648_ functionalized with specific ssDNA. When the target DNA is absent, the nanosensor is attached to a quencher. Binding with the target DNA leads to detachment of the nanosensor and recovery of fluorescence	Fluorescence detection	Excitation: 380 Emission (rpoB531): 535 Emission (katG315): 648	rpoB531: 24 pM; katG315: 20 pM	[91]
10	Blood	IFN-γ, IP-10	Aptamers specific for IFN-γ and IP-10	Cytosine–Ag^+^–cytosine and thymine–Hg^2+^–thymine hairpin structures releasing the metal ions upon specific interaction with different biomarker–aptamer complexes. Ag^+^ and Hg^2+^ are bound by CdTe and carbon QDs, which are detected by fluorescence	Fluorescence detection	-	IP-10: 0.3 × 10^−6^ pg/mL; IFN-γ: 0.5 × 10^−6^ pg/mL	[92]
11	Sputum	*M.tb* cells	*M.tb*-binding peptide H8, anti-*M.tb* polyclonal antibodies, and anti-HSP65 monoclonal antibodies	QDs conjugated with H8 or anti-HSP65 antibodies and MMS conjugated with H8 or anti-*M.tb* polyclonal antibodies. Magnetic separation of the QD–*M.tb*–MMS complex	Fluorescence detection (fluorescence microscopy)	Excitation: 405 Emission: 610	103 CFU/mL	[93]
12	*M.tb* suspension; sputum	*M.tb* cells	*M.tb*-binding peptide H8	Magnetic beads and QDs conjugated with H8. Magnetic separation of the QD–*M.tb*–magnetic bead complex	Fluorescence detection (fluorescence microscopy)	N/A	103 CFU/mL	[94]
13	Sputum	ESAT-6 gene	Oligonucleotides specific for ESAT-6 gene	FRET-based sandwich biosensor containing CdTe QDs and gold nanoparticles (quenchers) conjugated with the capture molecules (obtained by PCR). When the marker is bound, QD fluorescence is quenched via FRET to gold nanoparticles	Fluorescence detection	Excitation: 370 Emission: 400–680	10 fg	[95]
14	Sputum	*IS6110* DNA	ssDNA complementary to the *IS6110* gene fragment	FRET-based biosensor where CdTe QDs conjugated with the capture molecule serve as a donor and Cu-TCPP, which has a greater affinity for ssDNA than double-stranded DNA, serves as an acceptor. In the absence of the marker, the QD fluorescence is quenched. Hybridization of the ssDNA with the marker results in fluorescence, the intensity of which depends on the marker concentration	Fluorescence detection	Excitation: 365 Emission: 586	35 pM	[96]
15	Urine	Secretory antigen Ag85B	Anti-Ag85B antibodies (GBP-50B14 and SiBP-8B3)	FRET-based biosensor where gold nanorods conjugated with GBP-50B14 serve as acceptors and silica-coated CdTe QDs conjugated with SiBP-8B3 serve as donors. When both tags bind Ag85B, FRET between the QDs and nanorods quenches the QD fluorescence	Fluorescence detection	Excitation: 350 Emission: 630	13 pg/mL	[97]
16	Urine	LAM	Pair of anti-LAM recombinant monoclonal antibodies	Lateral flow test using CdSe/ZnS QDs encapsulated in polymeric beads conjugated with the capture molecules; test strip with the immobilized capture molecules	Portable fluorescence detector	Excitation: 375 Emission: 620	50 pg/mL	[98]
17	Urine	CFP-10	Pair of anti-CFP-10 antibodies	Glassy carbon electrode modified with graphene QD-coated Fe_3_O_4_@Ag nanoparticles and gold nanoparticles conjugated with the capture antibody. Binding of CFP-10 to the electrode results in an immune sandwich, gold nanoparticles conjugated with the detection antibody serving as signal-amplification labels	Differential pulse voltammetry	N/A	330 pg/mL	[99]
18	Exhaled air	TB-related volatile organic biomarkers	No	Suspension of CdSe or carbon QDs. The biomarker causes changes in the absorbance and fluorescence spectra	Spectroscopic analysis	Excitation: 360–650 Emission: 300–800	N/A	[100]
19	Exhaled air	MN	Co ion	CoTCPP nanosheets with attached CdTe QDs. The QD fluorescence is quenched in the absence of MN and is recovered upon MN binding to CoTCPP, causing QD release	Fluorescence detection	Excitation: 370 Emission: 658	0.59 µM	[101]
20	BALS; feces; paraffin-embedded tissues	*IS6110* and *IS900* DNA	*M.tb*-specific oligonucleotides	CdSe QDs conjugated with streptavidin and species-specific probes; magnetic beads conjugated with streptavidin and genus-specific probes. Sandwich hybridization is used to bind the biomarkers and subsequent magnet separation to concentrate the biomarker	Fluorescence detection	Excitation: 260 Emission: 655	12.5 ng	[102]
21	Pure fprA	fprA	Anti-fprA antibodies	Direct and double antibody sandwich lateral flow tests with CdSe/ZnS QDs conjugated with the capture molecule	Fluorescence detection	Emission: 565	12.5 pg/mL	[103]
22	*M.tb* strains	*M.tb* DNA	*M.tb*-specific ssDNA	FRET-based sensor composed of water-stable CsPbBr_3_ perovskite QDs conjugated to DNA probe serving as a donor and MoS2 nanosheets serving as an acceptor	Fluorescence detection	N/A	51.9 pM	[104]
23	Pure antigens	CFP10-ESAT6	Anti-CFP10–ESAT6 monoclonal antibody	Electrochemical immunosensor consisting of SPCE functionalized with Si nanoparticles and CdSe/ZnS QDs. The target biomarker is adsorbed on the electrode and then captured by the primary antibody, the secondary antibody being labeled with catalase, whose activity is detected electrochemically	Differential pulse voltammetry	N/A	15 pg/mL	[105]

Abbreviations: TMCC1, transmembrane and coiled-coil domain family 1; GBP6, guanylate binding protein family member 6; QD, quantum dot; IFN-γ, interferon gamma; TNF-α, tumor necrosis factor alpha; IL-2, interleukin-2; MAs, mycolic acids; CFP-10, culture filtrate protein 10; ssDNA, single-strand DNA; IP-10, IFN-γ-induced protein 10; MMS, magnetic microsphere; HSP65, heat shock protein 65; ESAT-6, early secretory antigenic target 6; FRET, Förster resonance energy transfer; Cu-TCPP, two-dimensional metal–organic framework; LAM, lipoarabinomannan; MN, methyl nicotinate; CoTCPP, cobalt-metalized tetrakis (4-carboxyphenyl) porphyrin; BALS, bronchoalveolar lavage specimens; fprA, flavoprotein reductase; SPCE, screen-printed carbon electrode; DPV, differential pulse voltammetry.

**Table 3:** Potential *M. tuberculosis* biomarkers.

Biomarker	Already Detected with QD-Based Nanosensors	Comment	Latent Form Detection	Ref.
**Host RNA Transcript/DNA Signatures**				
*GBP2*, *GBP5*, *GBP6*, *TMCC1*	+	Oligonucleotides (RNA, DNA)	+	[83,106]
*PRDM1*	−	PR domain zinc finger protein 1 gene	+	[106]
*ARG1*	−	Arginase 1 gene (encoding the arginase enzyme)	+	[106]
*IS6110*	+	*IS6110* gene	+	[96]
*IS1081*	−	*IS1081* gene	+	[107]
*rpoB531*	+	*rpoB531* gene	+	[91]
*katG315*	+	*katG315* gene	+	[91]
**Acids and their derivatives**				
MN	+	Menthyl nicotinate	−	[100]
MAs	+	Mycolic acids	+	[88,89]
**Enzymes**				
MNAzymes	+	Multicomponent nucleic acid enzyme	N/A	[87]
ADA	−	Adenosine deaminase (enzyme of purine metabolism)	+	[108]
KatGs	−	Catalase−peroxidase enzymes (responsible for the activation of the antituberculosis drug isoniazid)	−	[109]
**Сytokines**				
IL-1ra		Interleukin-1 receptor antagonist	−	[110]
IL-2	+	Interleukin-2	+	[110,111]
IL-10	+	Interleukin-10	+	[110,112]
IL-13		Interleukin-13	−	[110]
INF-y	+	Interferon gamma	+	[84,111,112]
TNF-α	+	Tumor necrosis factor alpha	+	[111]
INF-y IP-10	+	Interferon-gamma-inducible protein 10	+	[25]
MIP-1β	−	Macrophage inflammatory protein	N/A	[110]
**Specific surface proteins**				
CFP-10	+	10 kDa culture-filtered protein	+	[105,113,114]
Mtb Rv1468c (PE_PGRS29)	−	*M.tb* surface protein	N/A	[115]
Rv1509	−	*M.tb*-specific protein	N/A	[116]
ESAT-6	−	6 kDa early secreted antigenic target	+	[113,117,118,119]
MPT-64	−	*M.tb* protein 64	+	[120]
Ag85A, Ag85B	+	Secreted protein antigen 85 complex A & B	+	[97,121]
PPE-68	−	Proline–proline–glutamic acid	+	[122,123]
Rv2536	−	Potential membrane protein	+	[124]
Rv2341		Probable conserved lipoprotein LppQ	+	[125]
**Mycobacterial antigens**				
14 kDa antigen	−	14 kDa protein antigen	N/A	[126]
116 kDa antigen	−	*M.tb*-specific antigens	N/A	[127]
19 kDa antigen	−	19 kDa lipoprotein	+	[126]
30 kDa antigen	−	Immunodominant phosphate-binding protein	N/A	[128]
38 kDa antigen	−	Immunodominant lipoprotein antigen	N/A	[129]
55 kDa antigen	−	*M.tb*-specific antigens	N/A	[130]
LAM	−	A glycolipid and a virulence factor associated with *M.tb*	+	[131]
A60	−	Tuberculosis antigen	+	[132]
Mtb81	−	Recombinant protein	+	[133]
ESAT-6	+	*M.tb*-specific antigens	+	[105,134]
